# Morphological Study of Palatal Rugae in a Sudanese Population

**DOI:** 10.1155/2015/650648

**Published:** 2015-02-08

**Authors:** Altayeb Abdalla Ahmed, Awrad Hamid

**Affiliations:** ^1^Department of Basic Medical Sciences, College of Medicine, King Saud bin Abdulaziz University for Health Sciences, Mail Code 3127, P.O. Box 3660, Riyadh 11481, Saudi Arabia; ^2^Anatomy Department, Faculty of Medicine, University of Khartoum, P.O. Box 102, Khartoum 11111, Sudan; ^3^Department of Anatomy, Faculty of Dentistry, University of Medical Sciences and Technology, P.O. Box 12810, Khartoum 11111, Sudan

## Abstract

Palatal rugae patterns have unique characteristics and have been proposed as an alternative method to establish identity when other means, such as fingerprints and dental records, are not attainable. This study was conducted to determine the morphological characteristics of palatine rugae and to assess the existence of side asymmetry in them in Sudanese Arabs. It also assesses the possibility of determining sex using logistic regression. One hundred dental casts for 50 males and 50 females aged between 18 and 23 were studied for palatal rugae dimensions, shapes, and orientations, as well as sexual dimorphism and side symmetry. The most predominant rugae were primary, and the most prevalent shapes in both sexes were wavy, curved, and straight forms. The predominant orientation was forward. Side asymmetry existed more in the orientations than in the shapes, but no side asymmetry was recorded in the dimensions. There was no significant sexual dimorphism in the rugae dimensions, shapes, and orientations, except for forward-directed rugae (*P* < 0.037). A predictive value of 60% was obtained in assigning sex using dimensions and orientations and of 58% using shapes alone. Therefore, the palatal rugae are not recommended for assigning sex effectively among Sudanese Arabs unless it is the only means available.

## 1. Introduction

Palatal rugae (transverse palatine folds or palatine rugae) are anatomical structures present in the roof of the human mouth. Among humans, they are recognized as asymmetrical, irregular mucosal ridges arranged in a transverse direction on either side of the median palatine raphe in the anterior third of the palate behind the incisive papillae [1, 2].

Palatal rugae patterns are formed toward the third month of intrauterine life [3] and initially occupy much of the length of the palatal shelves at the time of their elevation [4]. Later, their pattern becomes less regular, with the anterior ones being more evident and compressed while the posterior ones disappear, resulting in their confinement to the anterior part of the secondary palate from the newborn stage onwards [2]. The regulation of the collagen fibers orientation in rugal morphogenesis has been attributed to the influence of certain genes [5].

Following palatal rugae formation, their length continues to change during palatine growth, but their position remains the same throughout life [6]. Therefore, it has been indicated that they gain their typical orientation pattern at birth and acquire their final shape in adolescence. Thereafter, they remain stable throughout life [7]. However, this stability of the palatal rugae patterns is a matter of considerable debate in the literature; some reports indicate that the rugae are closely related to teeth, following their movement after extraction of other teeth, resulting in a change in the original direction of the rugae [3]; in contrast, others indicate the retention of their stability [7, 8]. The more posterior rugae were suggested as being more stable [7]; however, it was also noted that the first rugae are the most stable [9]. Furthermore, pattern changes were observed following extreme finger sucking during infancy and orthodontic treatment due to persistent pressure [2, 10].

Palatal rugae shapes were proven to resist diseases, chemical aggression, and postmortem intrusions; additionally, they are well protected by the lips, a buccal pad of fat, and the teeth [11]. Previous reports assessed the influence of various factors on palatal rugae. It was found that they express less pronounced changes compared to the general condition of the victim of 3rd degree facial burns [12]. Despite the similarity in rugae patterns between twins, they are not identical, as indicted in Hauser et al. [4]. Therefore, the palatal rugae can be considered an ideal parameter for forensic identifications in terms of its uniqueness and postmortem resistance as well as stability following completion of growth [7, 13]. Moreover, they can be especially useful in cases where fingerprints cannot be attained, for example, when bodies are burnt or severely decomposed [12]. This makes postmortem identification possible in cases where there are antemortem records.

It is noteworthy that a comparison of rugae patterns between different ethnicities and within the subset of a single population showed diverse presentation patterns [2, 14–16]. Nonetheless, most earlier studies involved limited range of global populations. Palatal rugae morphological studies and systemic trends in precise sex determination are lacking among Sudanese Arabs. Sudan is a country with extensive indigenous diversity, and Sudanese Arabs form the largest ethnic group of contemporary Sudan and have a genetic mixture of Arabs and Africans [17, 18]. Hence, the present study aimed to determine the prevalence of the different biometric characteristics of the palatal rugae in the Sudanese Arab population, to explore the existence of asymmetry in these sexes, and to determine its effectiveness in identifying sexes using logistic regression.

## 2. Materials and Methods

### 2.1. Materials

The materials for this study included 100 dental casts, equally distributed between sexes. The age group involved in this study was between 18 and 23 years of age (the mean age of males was 21.76 ± 2.53, and the mean age of females was 21.33 ± 2.16). All participants were recruited from three universities in Khartoum. They were Sudanese Arabs with at least three generations on the mother's side and the father's side. Subjects with severe malocclusion, a history of palatal pathology, trauma or surgery to the palate, orthodontic treatment, active lesions, or any sign of allergy to impression material were excluded from the study. The study received approval from the ethical committee of the Faculty of Medicine, University of Khartoum, Khartoum, Sudan.

### 2.2. Methods

After obtaining the informed consent, a maxillary impression of the subject was taken using chromatic alginate impression material (Alginmax, MAJOR, Italy). The casts were immediately poured with improved type IV dental stone (Zhermack, Italy) to obtain greater strength and accuracy. All instructions by manufacturers were followed, including the water/powder ratio, vacuum mixing, and the use of a vibrator. Rugae patterns were delineated using a sharp 0.1 HB graphite pencil under adequate light and magnification utilizing a magnifying hand lens. All the rugae patterns were assessed and measurements were conducted by a single examiner (AH) using a digital sliding caliper and protractor.

**Figure 1:**
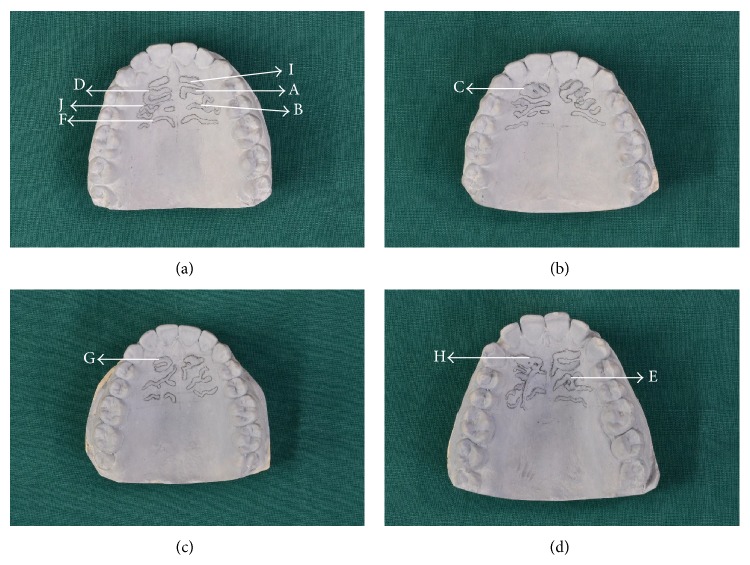
(a), (b), (c), and (d): different types of palatal rugae shape delineated in maxillary casts. A: angular, B: branching, C: circular, D: converging, E: crosslink, F: curved, G: diverging, H: nonspecific, I: straight, and J: wavy.

The rugae classification was recorded according to Lysell [7] and Thomas and Kotze [19, 20]. The palatine rugae lengths were measured on each side using a digital sliding caliper to an accuracy of 0.01 mm. Based on their length, the rugae were divided into three categories: primary rugae with lengths of more than 5 mm, secondary rugae with lengths from 3 to 5 mm, and fragmentary rugae with lengths between 2 and 3 mm. Rugae with lengths of less than 2 mm were disregarded. The shapes of the rugae (Figure 1) were classified into five major types: straight, curved, circular, wavy, and angular. On the other hand, the united rugae were categorized as follows: unification, branching, and crosslink. The unification was further subdivided into diverging and converging types. The branching type had branches that extended 1 mm or more from the origin in a lateral direction. Any rugae shape that did not fit into this classification was regarded as nonspecific. The direction of each primary ruga was classified according to the angle between the line joining its origin and termination with a line perpendicular to the median raphae. Forward-directed rugae were associated with positive angles. Backward-directed rugae were associated with negative angles, and perpendicular rugae were associated with no angle. The direction of the branched rugae was determined at their termination in the midpoint between the ends of the two branches.

### 2.3. Statistical Analysis

Prior to recording the findings, rugae observations were repeated twice for 20 casts to assess intraobserver error in interpretations. The discrepancies were not significant (*P* > 0.05). Association between length and directions and sex was assessed using the Mann-Whitney test. The association between rugae shape and sex was tested using chi-square analysis. Bilateral differences were assessed using Wilcoxon signed-rank tests. Logistic regression analysis (LRA) was used to assess the possibility of sex prediction utilizing a discrete variable (shape) and continuous variables (dimensions and orientation) using Statistical Package for the Social Sciences (SPSS), version 14 computer software (SPSS, Inc., Chicago, IL, USA).

## 3. Results

The prevalence and percentages of different rugae lengths in both sexes are described in Table 1. Males tend to have a larger number of rugae than females (665 and 621 rugae, resp.). Besides, the mean number of primary (505) and secondary (129) rugae in males was greater than that of females (486 and 103, resp.), whereas the fragmented rugae were comparable in both groups (31 to 32); however, there were no significant differences between sexes in the means. Primary rugae were predominant in both sexes compared to secondary and fragmented rugae.

When the shapes were considered (Table 2), in males curved rugae are the most prevalent form, followed by wavy rugae and then by straight rugae. In contrast, in females, wavy rugae are the most prevalent, followed by the curved rugae and then straight rugae. However, none of the forms showed any significant differences between the sexes (*P* > 0.05). The least common form in both sexes was the crosslink. Also, a few nonspecific rugae were observed. Unification constitutes approximately 5% of all rugae forms in males. The females tend to have more wavy shapes compared to the males (31.24% to 27.67%), whereas the males tend to have more curved and straight forms.

When we assessed the direction of the palatal rugae in both sexes (Table 3), the most common direction found in both sexes was forward, followed by backward and then perpendicular ones. Males tend to have more forward-directed rugae and fewer backward-directed rugae compared to females, but there are no statistically significant differences, except with forward-directed rugae (*P* = 0.037).

The bilateral differences in rugae lengths, shapes, and directions in both sexes were assessed and are summarized in Tables 4, 5, and 6. There was no significant asymmetry in all rugae lengths in both sexes, though the primary rugae were more on the left side in both sexes and secondary rugae tend to be more prevalent on the right side. The only significant differences in shapes between sides were found in the converging form in males (*P* < 0.039) and circular one in females (*P* < 0.013). Males showed significant asymmetry in rugae directions for the three shapes, whereas in females, the forward- and backward-directed rugae are significantly asymmetrical.

LRA was done for the combination of all shapes to assess the possibility of sex prediction. None of the shapes showed significant differences between sexes (Table 7). A predictive value of 58% was obtained in assigning the sex correctly (Table 8). When all continuous parameters (dimension and orientation) were used (Tables 9 and 10), none showed any significance, but the perpendicular orientation was excluded in the developed equation. However, sex prediction increased slightly to 60% with male prediction, better than with females (62% and 58%, resp.).

## 4. Discussion

Palatal rugae are a focus of interest due to their utilization in anatomy, anthropology, and genetics. In addition, they are important in biological profiling of individuals and can be an alternative method of identification when the application of conventional techniques, such as fingerprinting, dental recording, and DNA analysis, is not possible or limited [21, 22]. Moreover, palatine rugae have multiple uses in dental practice in terms of influencing prosthodontic treatment planes for edentulous patients, the diagnosis of submucosal clefts in the palate, and acting as reference points for measuring teeth migration during orthodontic therapy [1, 23, 24].

Various methods have been used to study palatal rugae patterns such as intraoral inspection, digital photography, stereoscopy, and stereophotogrammetry. In the present study, dental casts were used due to their simple analysis, easy manipulation, reliability, and the possibility of future comparative review, when needed. The age of the studied participants was selected due to the lack of an agreement on rugae stability with aging. While Lysell [7] indicated that the total number of rugae decreases after the age of 23 years, Yamazaki found that the mean ruga count changes moderately in adolescence but increases markedly from the age of 35 to 40 years, as discussed by Hauser et al. [4]. It has also been suggested that the characteristic pattern of palatal rugae remains stable from development up to seven days after death [11, 25]. According to Thomas and Kotze [19] there is no universally acceptable classification and the importance of successful identification exceeds the importance of the adopted classification; therefore, each examiner can use his own classification for comparative studies. In the current study, the method of rugae classification used [7, 19] was adopted as it is more practical than other methods, such as those followed by Hauser et al. [4] and Dawasaz and Dinkar [26]. It should be emphasized that no significant intraobserver errors were observed in this study (*P* > 0.05). The dimensions of the palatal rugae, which are continuous, are affected by growth and tooth extraction, whereas the discrete variable, shape, remains stable. Both were used and assessed by appropriate statistical methods. LRA can be used for continuous and discrete variables without the assumptions of a normal distribution, linearity, or equity of variance within each group [27]. Earlier authors indicated that using of nonparametric discriminant procedure is more appropriate [13, 27] than discriminant analyses determining ethnicity and sex.

The results of the present study showed that males have a larger number of rugae and more primary and secondary rugae than females. On the other hand, proportionally, the females showed more primary than secondary rugae compared to their male counterparts. The mean of the total number of rugae was 13.30 in males and 12.42 in females, which is greater than what was reported in Jordanians (8.8 in males and 8.5 in females) [28]. In addition, the mean of the total number of primary rugae was found to be 10.10 in males and 9.72 in females, which is similar to Australian Aborigines (10 for males and 9.8 for females) [2] but higher than in Rwandans (7.6 for males and 7.5 for females) [29], Saudis (7.3 for males and 7.2 for females) [30], Indians from Maharashtra (7.30 for males and 7.25 for females) [31], Caucasian Australians (8.6 each) [2], Indians (9.7 for males and 9.2 for females) [32], and Nepalese (8.9 for males and 8.5 for females) [32]. This variability between populations can be attributed to genetic and environmental factors. However, some authors have indicated clearly that environmental factors play a minimal role in affecting the formation of rugae and that the main determinant factor in the formation is the genetic background [2, 33].

These highlighted differences can also be attributed to differences in palatal width; it was indicated that differences in the mean number of rugae between populations are a reflection of greater ridge development, both qualitatively and quantitatively associated with the presence of broader palates [26] and that males show more primary and secondary rugae than their female counterparts which can also be attributed to the fact that males have larger palates than females, allowing them to have more lengthy rugae [26]. The statistical analysis used in this study did not reveal any significant differences between sexes in terms of the mean total number of all rugae dimensions, and this is in line with findings among Jordanians [28], Aboriginals [2], and some Indians [34, 35] but contradicts the findings in Western Indians [36].

In the present study the most common rugae shapes are wavy and curved forms, accounting for more than 55% of rugae in both sexes. Moreover, straight rugae constituted 22.54% and 24.06% in females and males, respectively. While the wavy shape was more common in females, the curved shape was slightly more common in males. These findings are in accordance with the findings in Egyptian and Saudi children [37], Saudi Adults [30], and Indians [32, 33, 38], where wavy and curved rugae were the predominant forms, followed by the straight one. Furthermore, they are consistent with the findings in Australian Aborigines and Caucasians populations [2], where wavy and curved rugae were found to form the majority. In contrast, in Jordanians [28], the most prevalent shape was wavy, followed by diverging, while in Rwandans [29] and Nepalese [32], the most prevalent shape was wavy, followed by straight and then curved ones. In our study, all the shapes were present. In contrast to Saudi children [37], who lack the crosslink shape, Sudanese Arabs have crosslink shapes similar to Egyptians children [37]. Nonetheless, it was the least recorded shape in both sexes. This variability in shapes between populations indicates the need for population-specific morphological studies to clarify the systemic trends among different populations. Also, the presence of some nonspecific rugae shapes may indicate the need for describing new rugae patterns in a larger sample. The findings of the present study indicate that there is no sexual dimorphism in rugae shapes (*P* > 0.05), which concurs with the findings for Jordanians [28] and Rwandans [29] and Egyptian and Saudi children [37] but contradicts the earlier findings in Saudi adults [30], where converging and circular patterns showed sexual dimorphism. This may suggest that either the degree of sexual differences in rugae patterns differs between populations or the differences expressed in some studies are individual-related variations.

The orientation of palatal rugae showed that the most prevalent direction is forward followed by backward and then perpendicular ones. Forward rugae are more common in males, and backward and perpendicular rugae are more common in females. This finding is consistent with the results obtained among Jordanians [28], but the reverse was reported among Rwandans [29], where females have more forward-directed rugae. Our results showed that there is a significant sexual difference in forward-directed rugae consistent with Jordanians [28].

On assessing bilateral differences, the present study showed that the left side tends to have more primary rugae in both sexes. This observation is similar to the findings in Australian Aborigines [2], Swedes [4], Rwandans [29], Mysoreans [33], and Japanese [39]. The right side was found to have more secondary rugae than the left side in both sexes. This observation gives credence to the assumption that there is an indirect relation between the primary and secondary rugae [26]. The findings of this study showed that there are no significant differences between the right and left sides in the means of the palatal rugae dimensions. Most of the shapes showed no significant bilateral differences, except for the converging shape in males (*P* = 0.039) and circular shape in females (*P* = 0.013). The orientations expressed the maximum degree of asymmetry in all directions in both sexes, except with the perpendicular direction in females. Forward-directed rugae were more on the left side in both sexes, whereas perpendicular and backward-directed rugae were more common on the right side in both sexes. This asymmetry in directions contradicts the assumption that the development of rugae is a coordinated process that occurs throughout the palate, indicating that differential growth rates exist between sides among sexes.

When the ability of palatal rugae to assign sexes was assessed using logistic regression analysis, the results obtained indicated that the shapes of the rugae are a weak discriminator between sexes in Sudanese Arabs (58% for both sexes). In contrast, the dimensions and directions yielded better prediction among males (62%), but female prediction did not improve. Previous studies among Indians allocated sex with a 99.2% success rate using shapes in 120 subjects using logistic regression [31], a 73.08% success rate using shapes and dimensions in 100 coastal Andhra Indian population, and a 78% success rate using dimensions only [35]. Another study among two groups of Indians showed no such sexual dimorphism [13]. When all continuous parameters were used, the assigning of sex among males improved slightly. Our findings revealed low success rate among Sudanese Arabs compared to the two previous mentioned studies; this may indicate ethnic variability in the expression of sexual dimorphism using palatal shapes as most of the previous studies among Indians indicated a lack of sexual dimorphism in palatal rugae, for example, that of Puducherry [34]. Moreover, this high success rate can be due to individual-related differences. Previous studies among Sudanese Arabs using limbs [40, 41] and crania [42] found moderate to high degrees of sexual dimorphism. The findings of the current study including palatal rugae dimensions, shapes, and directions did not show this discriminatory ability, which indicates that the palatal rugae cannot be used as an effective tool in assigning of sex.

Lastly, in the present study we used oral casts to assess morphological patterns which are two-dimensional; however, it is worthwhile assessing palatal rugae among Sudanese using other techniques, for example, stereoscopy and stereophotogrammetry to explore both rugae in three dimensions and the position of each ruga. The evaluation of casts was conducted by a single investigator as complex shapes may generate interobserver variation in identification [8]. Nevertheless, in spite of these shortcomings, this study has explored the utility of rugae as a biological profiling tool among the studied populations. It has also established a baseline data for any larger-scale study among the Sudanese that can be used for future comparative purposes among Sudanese or other populations with genetic mixtures between Arabs and Africans.

## 5. Conclusions

The palatal rugae dimensions, shapes, and directions were analyzed in a Sudanese population. The most predominant rugae were primary, and the most prevalent shapes found were the wavy, curved, and straight forms. The predominant direction was forward. There is a lack of any sexual dimorphism in the rugae dimensions and shapes, and the existence of sexual dimorphism was confirmed only in forward-directed rugae. The findings obtained showed that the use of palatal rugae to assign sex among Sudanese Arabs is not recommended. The possible differences in palatal rugae dimensions, shapes, and directions in different regions and even among different ethnicities with the same population necessitate further studies involving larger samples.

## Figures and Tables

**Table 1:** Descriptive statistics for palatal rugae lengths in both sexes.

Variable	Males				Females				Mann-Whitney test	
	Number	%	Mean	SD	Number	%	Mean	SD	*U*	*P* value
Primary	505	75.94	10.10	2.36	486	78.26	9.72	2.09	1127.00	0.392
Secondary	129	19.40	2.58	1.68	103	16.59	2.06	1.46	1035.50	0.131
Fragmented	31	4.66	0.62	0.83	32	5.15	0.64	0.75	1193.00	0.663

Total	665	100	13.30	2.47	621	100	12.42	2.85	975.00	0.056

SD: standard deviation.

**Table 2:** Descriptive statistics for palatal rugae shapes in both sexes.

Shape	Males				Females				Chi-square test		
	Number	%	Mean	SD	Number	%	Mean	SD	Chi-square value	Degree of freedom	*P* value
Straight	160	24.06	3.20	1.69	140	22.54	2.80	1.87	8.937	9	0.408
Curve	185	27.82	3.70	1.91	158	25.44	3.16	1.50	10.089	8	0.229
Wavy	184	27.67	3.68	1.56	194	31.24	3.88	1.53	7.429	7	0.378
Circular	16	2.41	0.32	0.59	15	2.42	0.30	0.58	0.174	2	1.000
Branch	40	6.02	0.80	0.93	35	5.64	0.70	0.74	2.133	3	0.585
Crosslink	5	0.75	0.10	0.30	9	1.45	0.18	0.39	1.329	1	0.249
Angular	30	4.51	0.60	0.81	27	4.35	0.54	0.93	2.849	4	0.690
Nonspecific	14	2.11	0.28	0.54	10	1.61	0.20	0.40	1.705	2	0.601
Converging	21	3.16	0.42	0.61	16	2.58	0.32	0.55	0.840	2	0.682
Diverging	10	1.50	0.20	0.45	17	2.74	0.34	0.63	2.289	3	0.659

**Table 3:** Descriptive statistics for palatal rugae directions in both sexes.

Direction	Males				Females				Mann-Whitney test	
	Number	%	Mean	SD	Number	%	Mean	SD	*U*	*P* value
Forward	389	58.50	7.78	3.16	319	51.37	6.38	2.59	949.500	0.037
Backward	231	34.74	4.62	2.57	247	39.77	4.94	2.65	1158.500	0.525
Perpendicular	45	6.77	0.90	1.18	55	8.86	1.10	1.25	1109.000	0.301

**Table 4:** Bilateral differences (left-right) in rugae lengths in both sexes.

Variable	Males			Females		
	Right mean ± SD	Left mean ± SD	*P* value^*^	Right mean ± SD	Left mean ± SD	*P* value^*^
Primary	5.02 ± 1.33	5.08 ± 1.65	0.842	4.76 ± 1.33	4.96 ± 1.34	0.299
Secondary	1.30 ± 1.04	1.28 ± 1.14	0.912	1.12 ± 1.04	0.94 ± 1.19	0.239
Fragmented	0.30 ± 0.46	0.32 ± 0.62	0.782	0.40 ± 0.70	0.24 ± 0.48	0.290

^*^Wilcoxon signed-rank test.

**Table 5:** Bilateral differences (left-right) in rugae shapes in both sexes.

Variable	Males			Females		
	Right mean ± SD	Left mean ± SD	*P* value^*^	Right mean ± SD	Left mean ± SD	*P* value^*^
Straight	1.62 ± 0.99	1.58 ± 1.30	0.684	1.38 ± 1.14	1.42 ± 1.31	0.944
Curve	1.94 ± 1.30	1.76 ± 1.35	0.396	1.72 ± 1.20	1.44 ± 1.05	0.258
Wavy	1.80 ± 1.20	1.88 ± 0.98	0.661	1.96 ± 1.14	1.92 ± 1.09	0.923
Circular	0.16 ± 0.37	0.16 ± 0.37	1.000	0.06 ± 0.24	0.24 ± 0.48	0.013
Branch	0.40 ± 0.67	0.40 ± 0.64	0.896	0.40 ± 0.57	0.30 ± 0.46	0.336
Crosslink	0.04 ± 0.20	0.06 ± 0.24	0.655	0.10 ± 0.30	0.08 ± 0.27	0.739
Angular	0.26 ± 0.53	0.34 ± 0.59	0.446	0.26 ± 0.63	0.28 ± 0.61	0.883
Nonspecific	0.14 ± 0.35	0.14 ± 0.41	1.000	0.12 ± 0.33	0.08 ± 0.27	0.527
Converging	0.12 ± 0.33	0.30 ± 0.51	0.039	0.16 ± 0.37	0.16 ± 0.42	1.000
Diverging	0.14 ± 0.35	0.06 ± 0.24	0.157	0.12 ± 0.48	0.22 ± 0.42	0.134

^*^Wilcoxon signed-rank test.

**Table 6:** Bilateral differences (left-right) in rugae directions in both sexes.

Variable	Males			Females		
	Right mean ± SD	Left mean ± SD	*P* value^*^	Right mean ± SD	Left mean ± SD	*P* value^*^
Forward	3.16 ± 1.89	4.62 ± 1.91	0.000	2.52 ± 1.87	3.86 ± 1.62	0.000
Backward	2.86 ± 1.63	1.76 ± 1.42	0.000	3.20 ± 1.91	1.74 ± 1.28	0.000
Perpendicular	0.60 ± 0.86	0.30 ± 0.65	0.022	0.56 ± 0.79	0.54 ± 0.76	0.885

^*^Wilcoxon signed-rank test.

**Table 7:** Regression coefficient of all shapes.

Sl. number	Variables	Regression coefficient (*b*)	*P* value
1	Straight	0.111	0.369
2	Curve	0.213	0.125
3	Wavy	−0.005	0.972
4	Circular	0.290	0.484
5	Branch	0.316	0.297
6	Crosslink	−0.598	0.343
7	Angular	0.061	0.806
8	Nonspecific	0.486	0.319
9	Converging	0.341	0.427
10	Diverging	−0.298	0.511
	Constant (*a*)	−1.475	0.247

**Table 8:** Predictive value using all shapes.

Sl. number	Actual group	Predicted value		Total number	Correct percentage
		Female (50)	Male (50)		
1	Female	29	21	50	58%
2	Male	21	29	50	58%
3	Overall				58%

**Table 9:** Regression coefficient of all dimensions and directions.

Sl. number	Variables	Regression coefficient (*b*)	*P* value
1	Primary	−0.071	0.707
2	Secondary	0.086	0.699
3	Fragmentary	−0.116	0.710
4	Forward	0.252	0.190
5	Backward	0.126	0.573
	Constant (*a*)	−1.804	0.107

**Table 10:** Predictive value using all dimensions and directions.

Sl. number	Actual group	Predicted value		Total Number	Correct percentage
		Female (50)	Male (50)		
1	Female	29	21	50	58%
2	Male	19	31	50	62%
3	Overall				60%
